# Examining Healthcare Professionals’ Telehealth Usability before and during COVID-19 in Saudi Arabia: A Cross-Sectional Study

**DOI:** 10.3390/nursrep12030064

**Published:** 2022-09-03

**Authors:** Mohammed Gh. Alzahrani, Nazik M. A. Zakari, Dina I. Abuabah, Mona S. Ousman, Jing Xu, Hanadi Y. Hamadi

**Affiliations:** 1 Department of Respiratory Care, College of Applied Sciences, AlMaarefa University, Diriyah, Riyadh 13713, Saudi Arabia; 2 College of Applied Sciences, AlMaarefa University, Riyadh 11597, Saudi Arabia; 3 Department of Health Administration, Brooks College of Health, University of North Florida, Jacksonville, FL 32224, USA

**Keywords:** COVID-19, telehealth, Saudi Arabia, interprofessionalism, usability

## Abstract

COVID-19 has placed substantial stress on healthcare providers in Saudi Arabia as they struggle to avoid contracting the virus, provide continued care for their patients, and protect their own families at home from possible exposure. The demand for care has increased due to the need to treat COVID-19. This pandemic has created a surge in the need for care in select healthcare delivery specialties, forcing other nonurgent or elective care to halt or transition to telehealth. This study provides a timely description of how COVID-19 affected employment, telehealth usage, and interprofessional collaboration. The STROBE checklist was used. We developed a cross-sectional online survey design that is rooted and grounded in the Technology Acceptance Model (TAM). The TAM model allows us to identify characteristics that affect the use of telehealth technologies. The survey was deployed in November 2021 to local healthcare providers in Saudi Arabia. There were 66 individuals in the final sample. Both interprofessional satisfaction on frequency and quality were positively correlated with the frequency of interactions. The odds for satisfaction of frequency and quality were about 12 times (OR = 12.27) and 8 times 110 (OR = 8.24) more, respectively, for the participants with more than three times of interaction than the participants with no interaction at all. We also found that change in telehealth usage during the pandemic was positively associated with the Telehealth Usability Questionnaire (TUQ) scores. The estimated score for the participants who reported an increase in telehealth usage was 5.37, while the scores were lower for the participants reporting ‘no change’ and ‘decreased usage’. Additional training on telehealth use and integration to improve interprofessionalism is needed.

## 1. Introduction

COVID-19 and related precautions have affected various healthcare services that have been either temporarily closed or transitioned to virtual care delivery and have placed substantial stress on healthcare providers. Therefore, using telehealth technologies and its policy incentivizing is an emergent need now more than ever. While work demands have varied, it is very important to examine the usability of telehealth implementation and services in Saudi Arabia during COVID-19. The combination of higher costs for the acquisition of personal protective equipment and an increased number of health providers testing positive has created a strain on the healthcare workforce [1]. Frontline healthcare providers have been reporting increased symptoms of anxiety and depression related to burnout, and fatigue and chronic concern for lack of personal protective equipment (PPE) [2]. The demand for care is relatively consistent; however, this pandemic has created a surge in the need for care in select healthcare delivery specialties, forcing other nonurgent or elective care to halt or transition to telehealth [3]. Even with the slowdown or in some cases halting of elective surgeries [4,5,6], millions of individuals with chronic diseases or non-COVID-19 illnesses still required access to care [4,7]. COVID-19 has created the need for ‘social distancing’ [8,9] to slow the spread of the virus, thus reducing the ability to provide in-person healthcare services. This forced distancing made telehealth an ideal modality to deliver necessary care [10]. Telehealth technologies group synchronous (phone and video) and asynchronous (store and forward such as patient portals) communication and virtual agents (telemonitoring through wearable devices); all of these activities allow the delivery of care and the interaction of provider-to-patient or provider-to-provider [11]. During these strenuous times, the need for telehealth services has pushed many organizations to expand their telehealth capabilities to serve patients while maintaining their safety at home [12].

However, most organizations and professions were not telehealth ready before the pandemic, causing staff resistance and lack of utilization of this technology within the interprofessional setting when it was needed most [13]. The lack of prior utilization of this technology is not the only challenge, but also several challenges are encountered in the usage and uptake of telehealth. These include digital illiteracy, technology, and internet access, gender, age, rural location, and low-income patients [14,15]. For telehealth to be effective during a healthcare crisis, we must rapidly understand how such technologies are being utilized and integrated into models of care. Therefore, this study aims to understand how healthcare providers, telehealth utilization, and interprofessional interactions were affected during COVID-19.

## 2. Materials and Methods

### Study and Instrument Design

We used a cross-sectional online survey design that is rooted and grounded in the Technology Acceptance Model (TAM). The TAM model allows us to identify characteristics that affect the use of telehealth technologies. Survey questions for this study were extracted from validated health surveys such as the Telehealth Usability Questionnaire (TUQ) [16,17,18]. This study expands on a previous study conducted in the United States and has been modified specifically for healthcare professionals in Saudi Arabia. The research team reviewed the survey instrument and ensured face validity. The study was submitted to the University Institutional Review Board (IRB), and received approval under ‘expedited review’. The final web-based survey consisted of demographic questions, provider practice questions, patient engagement questions, and the TUQ. We included questions on telehealth use before the start of the pandemic and during the third wave of the COVID-19 pandemic. This survey was created and disseminated using the web-tool Qualtrics™. Informed consent was at the beginning of the survey. Participants acknowledge a statement of consent to participate in the anonymous survey.

#### Setting

This study was conducted in hospitals in Saudi Arabia. Healthcare professionals employed and providing care to patients in Saudi Arabia were recruited for the study. The findings from this study will inform the factors impacting telehealth utilization and interprofessional interactions.

#### Participants

The inclusion criteria were for the healthcare professional to be employed and licensed. The survey was emailed to physicians, nurses, respiratory therapists, EMS specialists, social workers, physical therapists, and occupational therapists. Professions were identified based on the most common health providers for multidisciplinary healthcare work and involvement in COVID-19 treatment. Professionals were identified through snowballing sampling. The healthcare team’s local healthcare professional network was used to initiate the first responses and asked that the survey be shared with those individual colleagues from November 2021 until March 2022. Of those who opened the survey link, 63% completed the survey; there were 127 surveys started and 80 completed. Among the 66 individuals in the final sample, 50% were female, and 43.9% were citizens of Saudi Arabia. Regarding the primary profession, 66.7% were nurses, followed by physicians (18.2%), respiratory therapists (10.6%), and EMS specialists (4.6%). The majority of the participants (66.7%) had been licensed for less than 10 years, with each of the other categories (11–15 years, 16–20 years, and more than 21 years) representing about 33.3% of the sample (Table 1).

#### Data Analysis

Descriptive statistics were performed using SAS software Version 9.4.36. Descriptive statistics including means and frequencies were generated for participants (*n* = 66) who provided direct patient care during the COVID-19 pandemic. For the telehealth and interprofessional components of the survey, the summary was provided at both the overall level and expatriate status. To ensure homogeneity of the results, a sensitivity analysis was performed on a subset of the sample (*n* = 44) who were nurses and physicians.

## 3. Results

### 3.1. Satisfaction of Interprofessional Care Interactions

Of the 66 health professionals who participated in our survey 7.6% strongly agreed, 40.9% agreed, and 22.7% somewhat agreed that they were satisfied with the frequency of interprofessional care interaction 6 months before the pandemic (Table 2). In addition, 9.1% strongly agreed, 37.9% agreed, and 21.2% somewhat agreed that they were satisfied with the quality of interprofessional care interaction 6 months before the pandemic. When examining the time during the pandemic, 13.6% strongly agreed, 33.3% agreed, and 19.7% somewhat agreed that they were satisfied with the frequency of interprofessional care interaction. In addition, 15.2% strongly agreed, 31.8% agreed, and 16.7% somewhat agreed that they were satisfied with the quality of interprofessional care interaction. When we asked participants about telehealth usage for interprofessional collaboration during the COVID-19 pandemic compared to before the pandemic, 7 (10.6%) stated that their telehealth use decreased, 28 (42.4%) stated that their use did not change, and 31 (47%) stated that their telehealth use increased.

Statistical analysis was conducted to explore the satisfaction data further (Table 3). Both interprofessional satisfaction on frequency and quality showed no significant difference between 6 months before and during the pandemic. However, both satisfactions were positively correlated with the frequency of interactions. For example, the odds of satisfaction of frequency and quality were about 12 times (OR = 12.27) and 8 times (OR = 8.24) more, respectively, for the participants with more than three times of interaction than the participants with no interaction at all. The change in telehealth usage was the other significant factor.

### 3.2. Telehealth Usability

**Figure 1:**
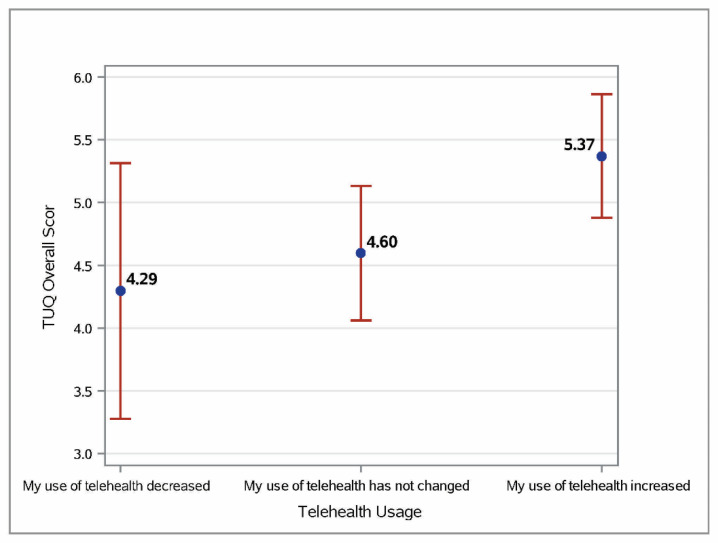
Least square mean estimates and 95% confidence interval for Telehealth Usability Questionnaire (TUQ) scores.

The only significant factor that impacted the overall Telehealth Usability Questionnaire (TUQ) scores was the change in telehealth usage during the pandemic (Figure 1). The estimated score for the participants who reported an increase in telehealth usage was 5.37, while the scores were 4.60 and 4.29 for the participants reporting ‘no change’ and ‘decreased usage’, respectively. The profession, age, gender, and participants from Saudi Arabia did not impact the overall TUQ score.

### 3.3. Subgroup Analysis

A subgroup analysis was also conducted to focus on the subgroup of nurses and physicians (44 in total). The results (not shown) for the satisfaction analysis were similar except that the change in telehealth usage was no longer significant. The results for the overall TUQ score showed there were no significant predictors. Both were possibly due to the reduction in sample size.

## 4. Discussion

To answer our research question, which is to understand how healthcare providers, telehealth utilization, and interprofessional interactions were impacted during COIVD-19, we found that both interprofessional satisfaction on frequency and quality were positively correlated with the frequency of interactions. Research has shown that incorporating interprofessional education can help improve both telehealth utilization and satisfy the need for and increase interprofessional interactions [19]. We also found that healthcare professionals in Saudi Arabia were more likely to use telehealth during the COVID-19 pandemic, according to our study’s findings. For telehealth to be effective during the current COVID-19 pandemic and future healthcare crises, we must understand how it is being utilized and integrated into evolving models of care, and examine the ability of health providers to use these technologies to improve the care delivery framework. This change not only creates an excellent opportunity for providers to demonstrate the value of telehealth, but also points to considerable education gaps that must be rapidly filled to take full advantage of the opportunity [20].

An array of free and commercial telemedicine applications (apps) has been created in Saudi Arabia in response to the COVID-19 outbreak. To ensure long-term viability of these services after the pandemic, it is vital to conduct usability testing of these apps. The healthcare organizations that provide telemedicine services in Saudi Arabia must be aware of the existing governing legislation and the accrediting authorities when developing telemedicine apps. During the pandemic, these organizations made several efforts to build and update their regulations to serve as a reference for healthcare providers and developers. Additionally, the Saudi Commission for Health Specialties has just launched a national online training course for healthcare providers to ensure a uniform approach to delivering telemedicine care. By making use of these tools, we can guarantee a high level of quality in telemedicine care as well as a satisfying user experience.

From a public health and health capacity standpoint, in Saudi Arabia, health authorities have been prepared to tackle any potential spread of infectious diseases associated with mass gatherings (e.g., the Hajj season). Currently, they implement the Ministry of Health (MOH) strategy for handling the disease. This strategy follows the Saudi Vision 2030 plan that stresses the importance of adopting and developing a national telehealth network to improve healthcare services accessibility across the kingdom. To screen suspected cases, provide long-distance care, and track COVID-19 patients, the Saudi MOH provided many telehealth mobile applications (e.g., Seha, Mawid, Tawakklna, Tabaud, and Tetamman) to be used instead of visiting primary care clinics. These telehealth tools were found effective in facilitating healthcare delivery, control the spread of COVID-19, and flatten the growth curve [21].

### Limitations

This study has several limitations that need to be recognized. First, this study was limited to a small sample of providers who are licensed in Riyadh, Saudi Arabia, so the results may not be generalizable to other care providers in other cities in Saudi Arabia or countries. Second, professionals’ main practice area was asked on the survey without a follow-up question on subspecialty or multispecialty; furthermore, only two main professions answered the survey.

## 5. Conclusions

This study sheds light on the utilization of telehealth and access to healthcare services. The result of the present study confirms the outcome of the previous studies [14,22,23]. There will be an increasing demand for initiatives to improve telehealth benefits or use among patients as more information about the benefits and consequences of using telehealth becomes available. Policymakers appeared to be reacting to the impending pandemic’s uncertainty as COVID-19 spread. Considering our findings, policymakers should think about making these temporary telehealth policies permanent in the event of another epidemic. To better prepare patients for future and unanticipated hurdles to in-person healthcare, authorities should consider adopting rules that favor the growth of telehealth services, such as specialized care. Owing to the potential benefits of telehealth services, policymakers should conduct additional analyses before devising strategies for managing future and unexpected obstacles that prevent patients from receiving in-person treatment.

## Figures and Tables

**Table 1:** Participants’ demographic characteristics, *n* = 66.

	*n*	%
Gender		
Male	33	50
Female	33	50
Expatriate		
No (Saudi citizen)	29	43.94
Yes (non-Saudi citizen)	35	50.03
No Response	2	3.03
Primary profession		
Physician	12	18.18
Nurse	44	66.67
Respiratory Therapist	7	10.61
Emergency Medical Services Specialist	3	4.55
Level of engagement in direct patient care		
<1 day per week	3	4.55
1–2 days per week	6	9.09
>2 days per week	57	86.36
Experience as a licensed provider		
0–5 years	27	40.91
6–10 years	17	25.76
11–15 years	13	19.7
16–20 years	6	9.09
21 or more years	3	4.55

**Table 2:** Satisfaction on interprofessional care interaction and telehealth usage, *n* = 66.

	6 Months before the Pandemic		During the Pandemic	
	Satisfied with Frequency	Satisfied with Quality	Satisfied with Frequency	Satisfied with Quality
Strongly disagree	6 (9.1%)	7 (10.6%)	7 (10.6%)	6 (9.1%)
Disagree	1 (1.5%)	1 (1.5%)	1 (1.5%)	2 (3.0%)
Somewhat disagree	5 (7.6%)	3 (4.6%)	7 (10.6%)	4 (6.1%)
Neither agree nor disagree	7 (10.6%)	9 (13.6%)	7 (10.6%)	11 (16.7%)
Somewhat agree	15 (22.7%)	14 (21.2%)	13 (19.7%)	11 (16.7%)
Agree	27 (40.9%)	25 (37.9%)	22 (33.3%)	21 (31.8%)
Strongly agree	5 (7.6%)	6 (9.1%)	9 (13.6%)	10 (15.2%)
No Response	0	1 (1.5%)	0	1 (1.5%)

**Table 3:** Impact of COVID-19 on interprofessional satisfaction, *n* = 66.

	Odds Ratio	Lower Confidence Level	Upper Confidence Level
Satisfaction on Frequency of Interprofessional Interaction			
>3 per day vs. no interaction	12.27	2.73	55.23
>3 per day vs. <1 per week	7.54	1.28	44.27
>3 per day vs. >1 per week	1.58	0.19	13.51
>3 per day vs. 1–2 per day	2.04	0.40	10.32
Satisfaction on Quality of Interprofessional Interaction			
>3 per day vs. no interaction	8.24	2.06	32.96
>3 per day vs. <1 per week	5.80	1.11	30.44
>3 per day vs. >1 per week	3.33	0.55	20.33
>3 per day vs. 1–2 per day	1.96	0.44	8.71

## Data Availability

The data that support the findings of this study are available on request from the corresponding author. The data are not publicly available due to privacy or ethical restrictions.
